# A phase 1b study of dual PD-1 and CTLA-4 or KIR blockade in patients with relapsed/refractory lymphoid malignancies

**DOI:** 10.1038/s41375-020-0939-1

**Published:** 2020-06-29

**Authors:** Philippe Armand, Alexander Lesokhin, Ivan Borrello, John Timmerman, Martin Gutierrez, Lili Zhu, Mihaela Popa McKiver, Stephen M. Ansell

**Affiliations:** 1 Department of Medical Oncology, Dana-Farber Cancer Institute, 450 Brookline Ave, Boston, MA 02215 USA; 2 Memorial Sloan-Kettering Cancer Center and Weill Cornell Medical College, New York, NY USA; 3 Johns Hopkins University, Baltimore, MD USA; 4 University of California Los Angeles, Los Angeles, CA USA; 5 Hackensack University Medical Center, Hackensack, NJ USA; 6 Bristol-Myers Squibb, Princeton, NJ USA; 7 Division of Hematology, Mayo Clinic, Rochester, MN USA

**Keywords:** Lymphoma, Lymphoma

## Abstract

Simultaneously targeting other pathways could increase the activity of PD-1 blockade in lymphoid malignancies not sensitive to single-agent blockade. We explored the safety and efficacy of combined PD-1 and CTLA-4 or KIR blockade in patients with relapsed/refractory (R/R) lymphoid malignancies. This phase 1b trial enrolled adult patients with R/R classical Hodgkin lymphoma (cHL), non-Hodgkin lymphoma (NHL), or multiple myeloma (MM). Patients received nivolumab plus ipilimumab (nivo/ipi) or lirilumab (nivo/liri) until complete response (CR), progression, or unacceptable toxicity. The primary endpoint was safety and tolerability, while secondary endpoints included overall (ORR) and CR rates (CRR), progression-free and overall survival. Sixty-five patients were treated with nivo/ipi, and 72 with nivo/liri. Twenty-nine percent of patients experienced grade 3–4 treatment-related adverse events with nivo/ipi, and 15% with nivo/liri. In cHL, ORR was 74% for nivo/ipi and 76% for nivo/liri, CRRs were 23% and 24%, respectively. In B-NHL and T-NHL, ORR range was 9–22% and CRR was 0–6%. No patient with MM had an objective response. While both combinations were active in cHL, the toxicity of nivo/ipi was higher than expected from nivolumab alone. These data suggest no meaningful improvement in the efficacy of the combinations over single-agent nivolumab in the diseases studied.

## Introduction

Programmed cell death-1 (PD-1) blockade has emerged as a powerful therapeutic tool in oncology, with successes and approved indications in a variety of solid tumor types. Nivolumab is an IgG4 monoclonal antibody (mAb) that binds to PD-1 and disrupts its interaction with its two ligands, PD-L1 and PD-L2. Nivolumab was tested in a phase 1 study in patients with relapsed/refractory (R/R) classical Hodgkin lymphoma (cHL), non-Hodgkin lymphoma (NHL), and multiple myeloma (MM; CheckMate 039, NCT01592370). The inclusion of patients with R/R cHL in a separate expansion cohort of CheckMate 039 followed the demonstration of a uniquely prevalent genetic abnormality at 9p24.1 in this disease, leading to overexpression of the genes for PD-L1 and PD-L2 [1] and overexpression of the corresponding proteins on the tumor cell surface [2]. This suggested a genetically determined vulnerability to PD-1 blockade in cHL. Indeed, the activity of nivolumab in cHL was robust, with a response rate of 87%, and a 6-month progression-free survival (PFS) of 86% [3]. This high activity was also seen in a phase 1 study of another anti–PD-1 mAb, pembrolizumab [4], and confirmed in two phase 2 studies [5–7], leading to FDA approval of these two agents for patients with R/R cHL. However, the activity of nivolumab in other tumor types, including follicular lymphoma (FL), diffuse large B-cell lymphoma (DLBCL), and MM, was much more limited [8]. Occasional responses were seen in B-cell NHL, but phase 2 studies in DLBCL [9] and FL (unpublished) did not confirm activity. Even in cHL, where nivolumab produced high response rates, the majority of patients progressed within 12–18 months on treatment [6]. This suggests the presence or development of resistance mechanisms to PD-1 blockade in all these tumor types, which could theoretically be overcome with combination therapy.

The present study was a continuation of CheckMate 039 testing two different combination strategies in cHL/NHL/MM. The first strategy was combined PD-1 and cytotoxic T-lymphocyte associated protein 4 (CTLA-4) blockade using nivolumab and the anti–CTLA-4 mAb, ipilimumab. CTLA-4 is another checkpoint pathway that may be usurped by human tumors, and in which blockade using a mAb can translate into therapeutic activity, as has been shown in melanoma [10]. Ipilimumab was used in a phase 1 study in NHL, which showed rare but durable responses in some patients, including those with FL and DLBCL [11]. Furthermore, animal models showed PD-1 blockade improves effector T-cell infiltration of tumors and response to CTLA-4 blockade against melanoma and colorectal tumors [12, 13], especially so with concurrent blockade, and that PD-L1 overexpression can be used by melanoma tumors as an escape mechanism to CTLA-4 blockade [14]. These preclinical lines of evidence highlight the possible synergy of combined targeting of CTLA-4 and PD-1, which has been demonstrated clinically in several solid tumors including melanoma [15, 16] and others [17, 18]. Based on this, we tested this combination in patients with cHL, NHL, and MM (nivo/ipi cohort).

The second strategy was to combine PD-1 blockade with killer cell immunoglobulin-like receptor (KIR) blockade. KIRs interact with human leukocyte antigen (HLA) molecules on the surface of cells to modulate the activity of natural killer (NK) cells, the principal effector cells of the innate immune system [19]. The KIR–HLA interaction provides self-tolerance against NK-mediated cytotoxicity. Its importance in antitumor immunity has been shown most convincingly in the context of allogeneic stem cell transplantation, where KIR-modulated NK alloreactivity can increase the graft-versus-leukemia effect [20, 21]. The potential therapeutic value of NK cell modulation in lymphoid malignancies is suggested by several lines of evidence. First, preclinical models suggested an important contribution of NK cells to the mechanism of action of PD-1 blockade [22] and demonstrated the activity of KIR blockade in murine lymphoma models [23]. Furthermore, cHL tumors frequently lack functional Class I and Class II major histocompatibility complex (MHC) molecules [24]. While the absence of functional MHC II does impair response quality and duration, nivolumab can still induce responses in tumors that lack both functional MHC I and II [25]. This strongly supports a potential role for NK cells in PD-1 blockade–mediated antitumor activity. Finally, a CD30–CD16A bispecific NK engager, AFM13, has shown single-agent activity in this disease, demonstrating the potential for NK-mediated cytotoxicity in cHL [26]. All together, these findings provide support for the use of NK-directed therapy in cHL. The fully human anti-KIR mAb, lirilumab, (BMS-986105, Innate Pharma, Marseille, France) was tested in a phase 1 study in patients with solid and hematologic malignancies, including 11 patients with indolent NHL. While there were no objective responses, the study demonstrated full KIR occupancy at all doses studied, continuous blockade at 3 mg/kg and above, and good tolerability with few severe adverse events (AEs) and no dose-limiting toxicity at doses up to 10 mg/kg. Here we examined the safety and preliminary efficacy of the combination of PD-1 and KIR blockade by concomitant administration of nivolumab and lirilumab (nivo/liri cohort).

## Subjects and methods

### Study design

This was a multicohort phase 1b clinical trial. The initial cohort, treated with nivolumab alone, has been described previously [27, 28]. Goal accrual for the nivo/ipi cohort was up to 75 patients (~14 with MM, ~32 with cHL or primary mediastinal B-cell lymphoma (PMBL), ~14 with B-NHL, and ~14 with T-NHL); 65 were actually enrolled; for the nivo/liri cohort the goal was up to 130 patients (~20 with MM, ~20 with cHL/PMBL, ~60 with B-NHL, and ~30 with T-NHL); 72 were actually enrolled. The study was conducted in accordance with the Declaration of Helsinki and with the Good Clinical Practice Guidelines of the International Conference on Harmonization. All patients provided written informed consent before enrollment. The study was registered at clinicaltrials.gov (NCT01592370).

### Outcomes

In both cohorts the primary endpoint was safety, defined as the number of patients with drug-related grade 3–4 AEs up to 100 days after the last dose of study drug. Principal secondary endpoints included overall response rate (ORR), best overall response, and PFS rate at 8, 16, and 24 weeks. Efficacy was separately assessed for cHL, B-NHL/PMBL, T-NHL, and MM patients. For systemic lymphomas, responses were assessed according to the International Working Group Revised Response Criteria [29]; for cutaneous T-cell lymphoma (CTCL), according to the Clinical End Points and Response Criteria in Mycosis Fungoides and Sézary Syndrome [28]; and for MM, according to the International Myeloma Working Group criteria [29]. Responses were assigned by investigators.

### Patients

The study population for both cohorts, like that of the original study [8], included patients ≥ 18 years old with R/R cHL, NHL, or MM, excluding Burkitt and lymphoblastic lymphoma. Patients with DLBCL had to have relapsed disease after autologous stem cell transplantation (ASCT) or failure of ≥ 1 prior multi-agent chemotherapy regimen in ASCT-ineligible patients; those with FL had to have had ≥ 2 prior lines containing rituximab and/or an alkylator; those with CD30+ anaplastic large cell lymphoma had to have prior treatment with brentuximab vedotin; and those with MM had to be refractory to ≥ 2 prior lines containing an immunomodulatory agent and a proteasome inhibitor; all others had to have received ≥ 1 prior treatment regimen. All patients had to have measurable disease, an Eastern Cooperative Oncology Group performance status (ECOG PS) of < 2, and adequate hematologic and organ function. Patients were excluded if they had active or prior central nervous system involvement; a concomitant second malignancy; prior allogeneic hematopoietic cell or solid organ transplantation; active or known autoimmune disease; prior treatment with a checkpoint blockade agent; or HIV, hepatitis B, or C infection.

### Procedures

In the nivo/ipi cohort, patients received nivolumab 3 mg/kg IV and ipilimumab 1 mg/kg IV on Day 1 of every 3-week cycle. After 4 cycles of combination therapy, nivolumab was continued alone at the same dose on Days 1 and 15 of every 4-week cycle. In the nivo/liri cohort, patients received nivolumab 3 mg/kg IV on Days 1 and 15, and lirilumab 3 mg/kg IV on Day 1 of every 4-week cycle. In both cohorts, treatment was continued for up to 2 years or until confirmed complete remission (CR), progressive disease (PD), or unacceptable toxicity. Patients with CR could continue treatment for the longer of an additional 16 weeks or until confirmation of CR on subsequent scheduled imaging assessment. Patients with PD could continue treatment if they appeared to derive clinical benefit, had stable ECOG PS, were not deemed at risk of serious complication, and provided informed consent to continue treatment. Treatment had to stop if further progression was confirmed upon subsequent imaging.

### Assessments

Safety was monitored continuously during the study and assessed using the National Cancer Institute Common Terminology Criteria for Adverse Events version 4.0. It is reported with all disease groups considered together for each cohort. Efficacy was assessed by disease-specific restaging. For lymphoma, patients were evaluated with computed tomography (CT) scans (and positron emission tomography as indicated); patients with CTCL were evaluated with CT scans and the modified Severity Weighted Assessment Tool; patients with MM were evaluated by monoclonal protein measurements in serum and urine or serum free light chains. These assessments were performed at baseline, after 2, 4, 6, and 8 cycles (nivo/ipi) or after 1, 2, 4, 6, and 10 cycles (nivo/liri), then in both cohorts every 4 cycles thereafter. Patients with MM were required to have a bone marrow biopsy at baseline and prior to Week 7, and additionally to document PD or CR.

### Statistical analysis

PFS was calculated for all patients from the date of first study treatment until progression or death from any cause, with patients censored at the last efficacy assessment date. PFS was estimated using the Kaplan–Meier method. Duration of response (DOR) was calculated from the date of documented response until progression or death, with patients censored at the last efficacy assessment date. The target confidence intervals (CIs) were calculated for the two cohorts and for each disease group, although this phase 1b study was not powered for efficacy endpoints. For example, anticipating ~14 patients per expansion cohort with nivo/ipi, if four responses were observed (29%), the lower limit of the 90% one-sided CI for the ORR would be 13%; anticipating ~20 patients per expansion cohort with nivo/liri, if five responses were observed (25%), the lower limit of the 90% one-sided CI for ORR would be 13%.

### Role of the funding source

The funder contributed to study design, data collection, data analysis, data interpretation, writing of the report (in addition to funding editorial assistance) and the decision to submit for publication. All authors had access to raw data, and the corresponding author had full access to all data in the study and had final responsibility for the decision to submit for publication.

## Results

In total, 65 patients were enrolled between April 2014 and July 2015 and treated in the nivo/ipilimumab cohort (Table 1): 31 with cHL, 16 with B-NHL (five with FL and 11 with DLBCL, including one with PMBL), 11 with T-NHL (including six with CTCL), and seven with MM. As expected, the cHL group was younger (median age 31–35 years). Patients received a median of six (range, 1–50) doses of nivolumab and four (range, 1–4) doses of ipilimumab. In the induction phase, 77% were treated with a relative dose intensity (RDI) ≥ 90% for nivolumab and 75% with an RDI ≥ 90% for ipilimumab. In maintenance, 84% had a nivolumab RDI ≥ 90%. At the time of database lock in May 2017, no patient remained on therapy.

**Table 1:** Baseline characteristics of the patients.

Variable	cHL	B-NHL	T-NHL	MM	Total
	Nivolumab + ipilimumab cohort				
Number of pts	31	16	11	7	65
Histology					
cHL	31				
FL		5			
DLBCL		11			
PMBL		1			
Systemic T-NHL^a^			5		
CTCL^b^			6		
MM				7	
Prior treatment					
Prior therapies					
Median (range)	4 (2–10)	3 (1–16)	4 (1–11)	5 (2–20)	4 (1–20)
Prior BV	30 (97)	1 (6)	4 (36)	0 (0)	35 (54)
Prior ASCT	12 (39)	1 (6)	1 (9)	5 (71)	19 (29)
Median age (range), years	35 (19–79)	66 (24–87)	56 (29–72)	64 (51–71)	51 (19–87)
Male	13 (42)	12 (75)	6 (55)	6 (86)	37 (57)
Female	18 (58)	4 (25)	5 (46)	1 (14)	28 (43)

	Nivolumab + lirilumab cohort				
Number of pts	21	32	9	10	72
Histology					
cHL	21				
FL		6			
DLBCL		26			
Systemic T-NHL^c^			6		
CTCL^d^			3		
MM				10	
Prior treatment					
Prior therapies					
Median (range)	4 (1–6)	3 (1–7)	2 (1–9)	6.5 (3–10)	3 (1–10)
Prior BV	18 (86)	0 (0)	2 (22)	0 (0)	20 (28)
Prior ASCT	2 (10)	5 (16)	3 (33)	4 (40)	14 (19)
Median age (range), years	31 (22–62)	62 (27–86)	70 (31–79)	58 (51–67)	56 (22–86)
Male	15 (71)	22 (69)	5 (56)	8 (80)	46 (64)
Female	6 (29)	10 (31)	4 (44)	2 (20)	26 (36)

Data are *n* (%) unless specified otherwise.

*ASCT* autologous stem cell transplantation, *B-NHL* B-cell non-Hodgkin lymphoma, *BV* brentuximab vedotin, *cHL* classical Hodgkin lymphoma, *CTCL* cutaneous T-cell lymphoma, *DLBCL* diffuse large B-cell lymphoma, *FL* follicular lymphoma, *MM* multiple myeloma, *NOS* not otherwise specified, *PMBL* primary mediastinal B-cell lymphoma, *pts* patients, *T-NHL* T-cell non-Hodgkin lymphoma.

^a^Including three with PTCL–NOS and two with other systemic T-NHL.

^b^Including one with Sézary syndrome, four with mycosis fungoides, and one with other CTCL.

^c^All with PTCL-NOS.

^d^All with mycosis fungoides.

In the nivo/liri cohort, 72 patients were enrolled between April 2015 and July 2016, and treated (Table 1): 21 with cHL, 32 with B-NHL (six with FL and 26 with DLBCL), nine with T-NHL (including three with CTCL), and 10 with MM. Patients received a median of five (range, 1–53) doses of nivolumab and three (range, 1–27) of lirilumab. Eighty-five percent had an RDI ≥ 90% for nivolumab and 85% for lirilumab. At database lock, two patients (5%) remained on therapy.

Among the 65 patients in the safety analysis for the nivo/ipi cohort, 91% had at least one grade 2 or higher AE and 63% at least one grade 3 or higher AE. There was one death not related to disease progression (septic shock, not related to study treatment). Seventy-nine percent of patients had at least one treatment-related AE (TRAE) of any grade, including 29% with at least one grade 3–4 TRAE (Table 2). There were no treatment-related deaths. The most common (> 10% patients) TRAEs of any grade were skin toxicity (including rash, dermatitis, dry skin, skin lesion, eczema, or pruritus; 28%), fatigue (26%), pyrexia (23%), diarrhea (19%), infusion-related reactions (IRR; 15%), cough (14%), nausea (14%), pneumonitis (12%), and arthralgias (11%). The most common (occurring in > 1 patient) grade 3–4 TRAEs were pneumonitis (5%), hyperlipasaemia (5%), hyperamylasemia (3%), vomiting (3%), increased alanine aminotransferase (3%), neutropenia (3%), and IRR (3%). Overall, 14 patients (22%) experienced at least one treatment-related serious adverse event (TR-SAE), including nine patients (15%) with at least one grade 3–4 TR-SAE: those included pneumonitis (*n* = 3), pneumonia (*n* = 1), febrile neutropenia (*n* = 1), nausea (*n* = 1), vomiting (*n* = 2), autoimmune pancreatitis (*n* = 1), diabetic ketoacidosis (DKA; *n* = 1), myasthenia (*n* = 1), and IRR (*n* = 1). In total, five patients (8%) stopped study treatment because of an AE (pneumonitis *n* = 4, DKA *n* = 1).

**Table 2:** Summary of treatment-related adverse events.

	Nivolumab + ipilimumab cohort, *n* (%)	Nivolumab + lirilumab cohort, *n* (%)
**Any grade (> 10% of patients in at least one cohort)**		
Fatigue	17 (26)	8 (11)
Pyrexia	15 (23)	2 (3)
Skin toxicity	18 (28)	17 (24)
Diarrhea	12 (19)	8 (11)
Nausea	9 (14)	3 (4)
Arthralgia	7 (11)	2 (3)
Pneumonitis	8 (12)	1 (1)
Cough	9 (14)	3 (4)
Infusion-related reaction	10 (15)	12 (17)
Total	51 (79)	51 (71)
**Grade 3–4 (occurring in any patient)**		
Fatigue	1 (2)	0
Diarrhea	1 (2)	0
Nausea	1 (2)	0
Vomiting	2 (3)	0
Autoimmune pancreatitis	1 (2)	0
Colitis	1 (2)	0
Increased lipase	3 (5)	1 (1)
Increased amylase	2 (3)	0
Increased alanine aminotransferase	2 (3)	0
Increased aspartate aminotransferase	1 (2)	0
Increased creatine phosphokinase	0	2 (3)
Lymphopenia	1 (2)	1 (1)
Neutropenia	3 (5)	3 (4)
Anemia	1 (2)	1 (1)
Febrile neutropenia	1 (2)	1 (1)
Myalgia	1 (2)	0
Pneumonitis	3 (5)	0
Pleural effusion	0	2 (3)
Hypophosphatemia	1 (2)	0
Hyperglycemia	1 (2)	0
Diabetic ketoacidosis	1 (2)	0
Dehydration	0	1 (1)
Infusion-related reaction	2 (3)	0
Cognitive disorder	1 (2)	0
Myasthenia	1 (2)	0
Pneumonia/lung infection	2 (3)	0
Tumor flare	0	2 (3)
Acute kidney injury	0	1 (1)
Total	19 (29)	11 (15)
Grade 5	0 (0)	0 (0)

Among the 72 patients in the safety analysis of the nivo/liri cohort, 82% had at least one grade 2 or higher AE, and 47% at least one grade 3 or higher AE. There were no deaths not related to disease progression. Seventy-one percent had at least one TRAE of any grade, including 15% with at least one grade 3–4 TRAE (Table 2). The most common (> 10%) TRAEs of any grade were skin toxicity (24%), IRR (17%), fatigue (11%), and diarrhea (11%). The most common (> 1 patient) grade 3–4 TRAEs were increased creatinine phosphokinase (CPK; 3%), neutropenia (3%), pleural effusion (3%), and tumor flare (3%). Overall, four patients (6%) experienced a TR-SAE, including three (4%) with a grade 3–4 SAE: these included two (3%) tumor flares and one each of pleural effusion, febrile neutropenia, and acute kidney injury. No patient in this cohort stopped study treatment because of an AE.

**Fig. 1:**
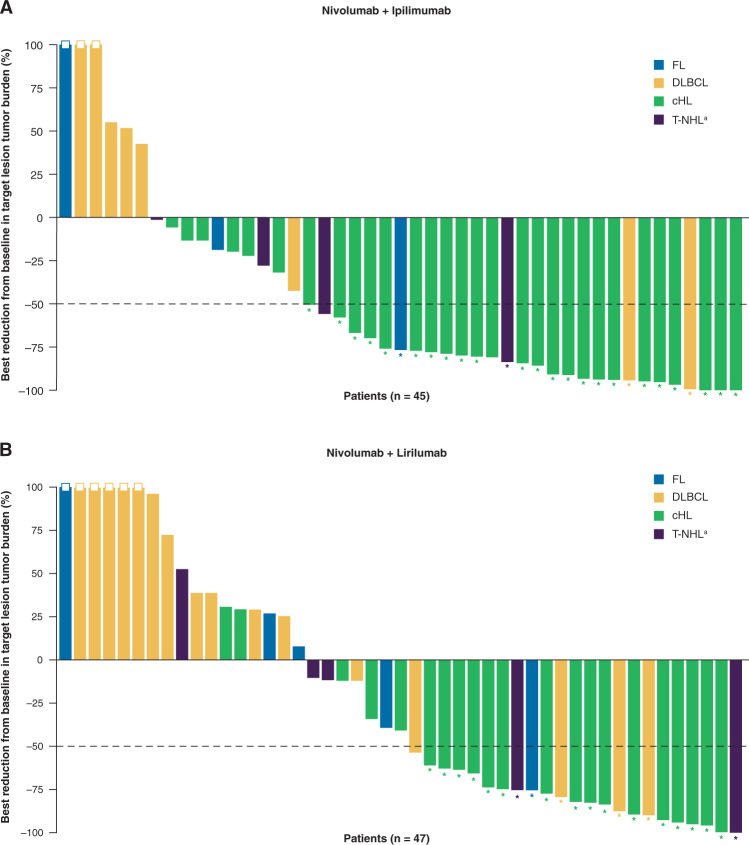
Change in measurable disease at best response for all evaluable patients, excluding patients with multiple myeloma. **a** Nivolumab + ipilimumab cohort, **b** nivolumab + lirilumab cohort. ^a^Excluding patients with cutaneous T-cell lymphoma. Asterisk indicates responders; box indicates tumor burden truncated to 100%. *cHL* classical Hodgkin lymphoma; *DLBCL* diffuse large B-cell lymphoma; *FL* follicular lymphoma; *T-NHL* T-cell non-Hodgkin lymphoma.

**Fig. 2:**
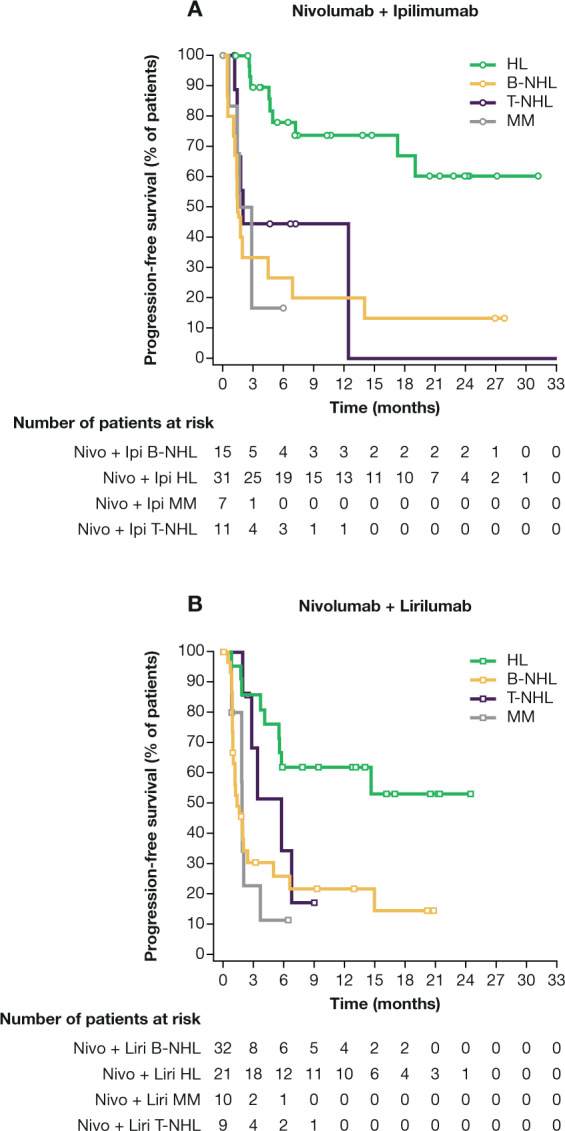
Progression-free survival. **a** Nivolumab + ipilimumab cohort, **b** nivolumab + lirilumab cohort. *B-NHL* B-cell non-Hodgkin lymphoma; *HL* Hodgkin lymphoma; *ipi* ipilimumab; *liri* lirilumab; *MM* multiple myeloma; *nivo* nivolumab; *T-NHL* T-cell non-Hodgkin lymphoma.

Response rates of the nivo/ipi cohort are summarized in Table 3. Among patients with cHL, ORR was 74%, including 23% who achieved CR as best response. Fifty-eight percent of responses were obtained within 4 months and 36% within 9 weeks. There was no obvious association between response and baseline characteristics such as time from diagnosis (38% of non-responders were within 2 years of diagnosis versus 30% of responders), age (median age 40 years [range 21–79] in non-responders versus 35 years [range 19–71] in responders), or number of prior therapies (median 3 [range, 2–7] in non-responders versus 4 [range, 2–10] in responders). Among patients with B-NHL, ORR was 19% and CR rate (CRR) was 6% (for FL, ORR was 20% and CRR was 0%; for DLBCL, ORR was 18% and CRR was 9%). Among patients with T-NHL, ORR was 9%, with no CRs. Change in measurable disease at best response for all evaluable patients with cHL and NHL is shown in Fig. 1a. Among patients with MM, there were no objective responses (ORR was 0%). With a median follow-up for survivors of 18 months, the median PFS among patients with cHL was not reached (95% CI, 17 months to not reached; Fig. 2a); the median DOR was not reached (95% CI, 16 months to not reached). Among all other cohorts, the median PFS was 1–2 months (Table 3). Among all 65 patients, 30 (46%) died, including six (9%) who died within 30 days of the last dose of study treatment. Most deaths were due to PD, except two (one from cytomegalovirus infection and septic shock and one from hepatic failure).

**Table 3:** Summary of response rates and progression-free survival by cohort and disease.

	Nivolumab + ipilimumab cohort				Nivolumab + lirilumab cohort			
	cHL	B-NHL	T-NHL	MM	cHL	B-NHL	T-NHL	MM
*n*	31	16	11	7	21	32	9	10
BOR	74% (55–88)	19% (4–46)	9% (0–41)	0% (0–41)^a^	76% (53–92)	13% (4–29)	22% (3–60)	0% (0–31)^b^
PR	52% (33–70)	13% (2–38)	9% (0–41)		52% (30–74)	9% (2–25)	22% (3–60)	
CR	23% (10–41)	6% (0–30)	0% (0–29)		24% (8–47)	3% (0–16)	0% (0–34)	
SD	13%	13%	55%		14%	13%	22%	
PD	10%	44%	18%		10%	50%	33%	
NA^c^	3%	25%	18%		0%	25%	22%	
PFS (months)								
Median	NR (17–NR)	1 (0–5)	2 (1–12)	2 (1–NC)	NR (6–NR)	1 (1–2)	6 (2–NC)	2 (1–4)
12 months	NC	19% (5–40)	NC	0	62% (38–79)	22% (9–39)	NC	0
DOR (months)								
Median	NR (16–NR)	NR (11–NR)	NR	NC	NR (14–NR)	NR (NR–NR)	3 (3–3)	NC

Response rates are provided together with 95% confidence intervals. PFS and DOR are provided as median with 95% confidence intervals in parentheses.

*B-NHL* B-cell non-Hodgkin lymphoma, *BOR* best overall response, *cHL* classical Hodgkin lymphoma, *CR* complete remission, *DOR* duration of response, *MM* multiple myeloma, *NA* not applicable, *NC* not calculated, *NR* not reached, *PD* progressive disease, *PFS* progression-free survival, *PR* partial remission, *SD* stable disease, *T-NHL* T-cell non-Hodgkin lymphoma.

^a^Three patients were not evaluable for response.

^b^Two patients were not evaluable for response.

^c^Includes patients who died or stopped for toxicity prior to first assessment.

In the nivo/liri cohort, ORR, and CRR, respectively, were 76 and 24% for cHL. Here again we detected no clear association between response and baseline characteristics including time from diagnosis (60% of non-responders were within 2 years of diagnosis, versus 56% of responders), age (median age 29 years [range 22–55] in non-responders versus 33 years [range 22–62] in responders), or number of prior therapies (median 4 [range, 3–6] in non-responders versus 4 [range, 1–5] in responders). ORR and CRR in NHL were: 13 and 3% for B-NHL (17 and 17% for FL, 12 and 0% for DLBCL), 22 and 0% for T-NHL, and 0% for MM (Table 3). Change in measurable disease at best response for all evaluable patients with cHL and NHL is shown in Fig. 1b. With a median follow-up for survivors of 11 months, the median PFS among patients with cHL was not reached (95% CI, 6 months to not reached; Fig. 2b); the median DOR was not reached (95% CI, 14 months to not reached). Among patients with B-NHL or MM, the median PFS was 1–2 months, and among those with T-NHL was 6 months (Table 3). Among all 72 patients, 34 (47%) died, including three (4%) who died within 30 days of the last dose of study treatment, again with most deaths due to PD except one (from neutropenic sepsis).

## Discussion

In this extension of the original phase 1 study of nivolumab in lymphoid malignancies, we sought to explore the safety and efficacy of combined checkpoint blockade using PD-1 blockade as a foundation and adding either CTLA-4 or KIR blockade. Overall, the results demonstrate that those drug combinations, specifically nivolumab + ipilimumab and nivolumab + lirilumab, are generally tolerable in patients with advanced cHL, NHL, and MM. In patients treated with nivo/liri, there was no evidence of significant toxicity increase compared with the extensive experience in hematologic malignancies with single-agent nivolumab [3, 6, 8]. However, as expected from comparable experience in solid tumors [15], the toxicity of nivo/ipi appeared higher than that of nivolumab alone, with nearly one-third of patients experiencing a serious TRAE, distributed across a range of systems (Table 2).

Interpretation of the efficacy data requires caution. In all cases, it is important to interpret safety and efficacy results while considering the results of single-agent treatment, which are very different across the histologies studied here. The eligibility criteria for the two cohorts described here were the same as those of the previously published single-agent cohort [3, 8], and the present study was conducted within a subset of the participating centers in the original phase 1 study. However, this study was not powered for efficacy, and comparisons between the outcomes of the two cohorts presented here and those of prior cohorts of patients treated with single-agent nivolumab should remain exploratory. Also, the individual cohorts of specific NHL subtypes are too small to draw definitive conclusions about the specific efficacy of the nivolumab combinations within any of those subgroups.

In the case of cHL, the single-agent activities of ipilimumab and lirilumab have not yet been tested. PD-1 blockade is very active; in large phase 2 studies of nivolumab and pembrolizumab, ORR was around 70%, CRR around 20%, and median PFS around 1 year [5, 6]. This provides a robust comparator for response and response duration. The ORRs with nivo/ipi (74%) and nivo/liri (76%) and CRRs (23% and 24%, respectively) do not seem different enough from those expected with PD-1 blockade to be clinically relevant. The PFS with nivo/ipi in cHL may be superior to that of single-agent nivolumab (median PFS with nivolumab 15 months versus not reached with nivo/ipi in this study). However, this comparison is limited by the small sample size. It therefore does not appear that the addition of CTLA-4 or KIR blockade increases either the frequency or depth of responses. However, it is possible that the nivo/ipi combination could provide a PFS benefit, which will require longer follow-up to evaluate.

In NHL, biological heterogeneity across and within disease types presents a significant challenge in small phase 1 studies, which may not be large enough to identify important treatment effects in selected subsets of NHL reliably. Nonetheless, the response rates noted here with nivo/ipi and nivo/liri in B-NHL and T-NHL (Table 3) do not seem appreciably different from each other or from those seen with single-agent PD-1 blockade. Certainly specific tumor subtypes within those categories could potentially have increased sensitivity to one or the other combination, but our study would not permit such exploration. In the case of MM, the absence of any objective response with either combination argues against any therapeutic benefit of these drugs in an unselected MM patient population. For all of these diseases, it remains to be understood what the immune resistance pathways are in those diseases and whether they can be modified to therapeutic benefit with agents that target other pathways. This will require more detailed investigations of the immune architecture of the tumor microenvironment and of the biological characteristics of the rare responding patients. Such insights may come from analyzes of other ongoing clinical trials of single-agent checkpoint blockade in NHL, including phase 2 studies of PD-1 blockade in DLBCL and FL (e.g., NCT02038946, NCT02038933, NCT03586024, and NCT03316573).

In conclusion, while combining PD-1 and CTLA-4 blockade (nivo/ipi) or PD-1 and KIR blockade (nivo/liri) is feasible in patients with advanced lymphoma and MM, the combination of nivo/ipi appears to be associated with increased but manageable toxicity over that expected with single-agent nivolumab. Furthermore, the present data do not suggest that those combinations meaningfully improve the already strong therapeutic activity of nivolumab in cHL, nor do they suggest that the combinations provide a significant therapeutic benefit in unselected B-NHL, T-NHL, or MM populations, again emphasizing that this conclusion relies on a small number of patients. These results may inform the design of future checkpoint-based trials, as they suggest that the simultaneous blockade of two inhibitory immune signals in lymphoid malignancies may not be effective, unlike in some solid tumors; future strategies could focus on the combination of checkpoint inhibitor with agents that directly deplete tumor cells (such as antibody–drug conjugates or cytotoxic chemotherapy) or agents that activate the immune system (agoniztic antibodies, vaccines, chimeric antigen receptor T cells, etc.).

### Data sharing

The BMS policy on data sharing may be found at https://www.bms.com/researchers-and-partners/independent-research/data-sharing-request-process.html.
